# Kienböck’s Disease – Incidence and operations in Finland: a nationwide register study

**DOI:** 10.1177/17531934241312894

**Published:** 2025-01-24

**Authors:** Krista Wernér, Turkka Anttila, Timo Viljakka, Jorma Ryhänen, Sina Hulkkonen

**Affiliations:** 1 Musculoskeletal and Plastic Surgery, Department of Hand Surgery, University of Helsinki and Helsinki University Hospital, Helsinki, Finland; 2 Tampere University Hospital, Tampere, Finland

**Keywords:** Kienböck’s disease, osteonecrosis, lunate bone, epidemiology, incidence, surgical procedure

## Abstract

This study assessed the nationwide incidence of Kienböck’s Disease, and operations performed for Kienböck’s Disease from the Care Register for Health Care in Finland 1996–2022. The mean annual total standardized incidence rate per million person-years was 9.60 (95% CI: 9.10–10.11) and for men and women, 10.38 (95% CI: 9.63–11.13) and 8.78 (95% CI: 8.10–9.45), respectively. The incidence rate was highest for 50–59-year-olds, at 15.49. Compared with women, the incidence rate for men was higher across all age groups up to 60–69 years. Altogether, 44% of the patients were operated on, and 19% of them had multiple operations. Partial arthrodesis was the most performed procedure and salvage procedures covered 62% of all operations. Our findings provide comprehensive new evidence about Kienböck’s Disease distribution across Finland’s population and offer insights into the burden caused by operations.

**Level of evidence:** III

## Introduction

There is little published data on the epidemiology of Kienböck’s Disease’s (KD) (Golay et al., 2016; van Leeuwen et al., 2016; Mennen and Sithebe, 2009; Tsujimoto et al., 2015) but it is considered an uncommon disorder. The reported prevalence of asymptomatic cases varies from 0.0066% to 1.9% (Golay et al., 2016; van Leeuwen et al., 2016; Mennen and Sithebe, 2009; Tsujimoto et al., 2015). According to previous studies, men seem to be most susceptible to KD, especially 20–40-year-olds, but research on this is scarce, and the study populations are small (Daly and Graf, 2022; Rioux–Forker and Shin, 2020; Schuind et al., 2008).

The natural progression of KD may vary considerably (Hwang et al., 2022; Kristensen et al., 1986; Viljakka et al., 2016). Clinical manifestation, from the start of the symptoms to arthritis, can take from months to decades (Martini, 1990; Stahl et al., 2014). Also, several studies have reported that the radiological stage may deteriorate without necessarily having a clinical impact (Degeorge et al., 2021; Van den Dungen et al., 2006; Taniguchi et al., 2003; Viljakka et al., 2016). The current understanding is that the natural course is more benign in children and in people aged over 70 (Irisarri et al., 2010; Lichtman et al., 2017; Taniguchi et al., 2003). Recently, Hwang et al. 2022) suggested a more benign disease course in patients aged over 50.

Numerous surgical procedures have been proposed and used as a treatment for KD. Nevertheless, the current literature fails to prove the superiority of any operation (Ho Shin et al., 2018; Innes and Strauch, 2010; Martin and Squire, 2013; Park et al., 2023). Furthermore, no research to date has determined whether any treatment can affect the natural progression of KD (Ho Shin et al., 2018; Innes and Strauch, 2010; Martin and Squire, 2013; Park et al., 2023; Wang et al., 2020).

To our knowledge, no population-based studies have examined the incidence, epidemiology, and surgical procedures performed for KD. Thus, there is uncertainty about its total, age- and sex-related incidence and the surgical procedures used in clinical settings.

This study aimed to determine the incidence rate of KD in Finland, to assess the surgical procedures that are used for KD and to determine how many patients undergo several operations. We also analysed whether there were trends in some combinations of consecutive operations. We conducted a retrospective cohort study based on data from the Care Register for Health Care controlled by the Finnish Institute for Health and Welfare (THL).

## Methods

The Finnish Social and Health Data Permit Authority (Findata) assessed and processed our register data request and approved the publication of the results (THL/2563/14.02.00/2022). The data of diagnoses and procedures were collected between 1 January 1996 and 31 December 2022 from the Care Register for Health Care, which is a nationwide register covering all of Finland. KD patients were identified using the International Classification of Diseases (10th revision, ICD–10) codes M93.1 (KD in adults) and M92.2 (Juvenile osteochondrosis, hand). All the surgical procedures performed with KD as a main diagnosis were obtained from the register. Finnish registers have been shown to be comprehensive and reliable in international comparisons, and hence enable epidemiologic studies of high quality (Sund, 2012). After collecting patient data from the register, Findata pseudonymized the patients and delivered the data to a secure operating environment. To calculate incidences, we obtained the Finnish population structure statistics in age groups and stratified by gender from Statistics Finland (https://www.tilastokeskus.fi).

We included patients who had been diagnosed when they were over 5 years of age. We calculated the crude incidence rate of KD by dividing the number of new cases by the population in Finland during the same period. We present it as patients per million person-years (ppm), calculated separately for men and women, and in different age groups. We also calculated the standardized incidence rates to make the comparison of our findings with different population demographics easier. We did this using the direct method in 5 year age groups as a standard with the 2013 European Standard Population (https://ec.europa.eu/eurostat/web/products-manuals-and-guidelines/-/ks-ra-13-028). Finally, we calculated the 95% confidence intervals (CI) assuming that the number of cases followed the Poisson distribution.

The surgical procedures were coded according to the Nordic Medico-Statistical Committee (Nomesco) procedural classification. All the authors of this article agreed on the relevant surgical procedure codes from the data and on their classification. We used two categories: the type of the surgical procedure (eight classes); and salvage vs. non-salvage procedures. The classification of the surgical procedures is presented in Table 1. The surgical procedures that included removing the lunate were considered to be salvage procedures. The age demographics of the operated patients were obtained at the time of the first operation. In some cases, one operation could include multiple surgical procedure codes. For combined operation calculations, we used a separate prioritizing command to filter the assumed main procedure for each operation. The priorities, from high to low, were: vascularized bone grafts; total arthrodesis (TWA); partial arthrodesis (PWA); prosthesis; proximal row carpectomy (PRC)/removal of the lunate; osteotomies; unclassified; and debridement. Determination of prioritization was based on the expert opinion and clinical experience of the authors. Statistical significance for the sex-wise distribution to salvage and non-salvage operations was calculated with the chi-squared test. A *p-*value <0.05 was considered significant. The data were analysed using RStudio Software version 4.3.1.

**Table 1.** Surgical procedures and their classification according to the Finnish version of the Nordic Classification of Surgical Procedures.

	Code	Definition	Salvage/non-salvage
Osteotomy	NCK30	Angulation, rotation or displacement osteotomy of elbow or forearm, other location	Non-salvage
	NCK68	Shortening or lengthening osteotomy of elbow or forearm, other location	
	NDK30	Osteotomy and rotation of a bone in the hand or wrist	
	NDK68	Shortening or lengthening of a bone in the hand	
	NCK70	Bone transport operation of elbow or forearm, other location	
Vascularized bone graft	ZZQ40	Free microvascular graft of bone	Non-salvage
	YNA05	Excision of bone with nutrient vessels for transplantation	
	ZZR45	Skeletal flap	
	ZZS20	Distant flap	
Debridement	NDF20	Operation for osteochondritis of joint in the wrist, open	Non-salvage
	NDF25	Operation for osteochondritis of joint in the wrist, arthroscopic	
	NDF10	Synovectomy of the wrist	
	NDF15	Synovectomy of the wrist, arthroscopic	
PRC/removal of the lunate	NCK10	Partial or total excision of bone in the elbow or forearm, other location	Salvage
	NDK00	Excision or incision of a bone in the wrist	
	NDK10	Incision or excision of a bone in the hand	
	NDG39	Other excision, reconstruction or fusion of joint in the wrist	
	NDG00	Arthroplasty of wrist	
Prosthesis	NDB10	Primary partial prosthetic replacement of joint in the wrist not using cement, other or unspecified	Salvage
	NDB20	Primary total prosthetic replacement of joint in the wrist not using cement, all parts	
	NDC20	Secondary implantation of partial prosthesis in joint of the wrist	
	NDC99	Other secondary prosthetic replacement in joint of wrist or hand, other or unspecified	
	NDH30	Reduction of dislocation of joint prosthesis of wrist, closed	
	NDU00	Removal of prosthesis from wrist	
	NDU99	Removal of other implant from wrist or hand	
Partial arthrodesis	NDG20	Fusion of wrist, partial	Salvage
	NDK99	Other operation on bone in the hand or wrist	
Total arthrodesis	NDG30	Fusion of wrist, total	Salvage
Unclassified	NCK99	Other operation on bone in the elbow or forearm, other location	Non-salvage
	ACC19	Transcision of peripheral nerve: other or unspecified	
	NDF99	Other operation on synovia or articular cartilage of the wrist	
	NDH99	Other operation on joint in the wrist or hand, unspecified	
	NDT99	Other operation on wrist or hand	

PRC, Proximal row carpectomy; salvage, surgical procedures including removal of the lunate.

## Results

The mean annual total standardized and crude incidence rate for KD in Finland was 9.60 ppm (95% CI: 9.10–10.11) and 9.65 over the study period, respectively. The standardized and crude incidence rates for men and women were 10.38 (95% CI: 9.63–11.13), 10.70, 8.78 (95% CI: 8.10–9.45) and 8.63, respectively. We found that the highest incidence rate per age group was in the 50–59 age group, at 15.49 ppm, and the lowest was in the 0–9 year age group, at 0.50 ppm.

**Figure 1.**
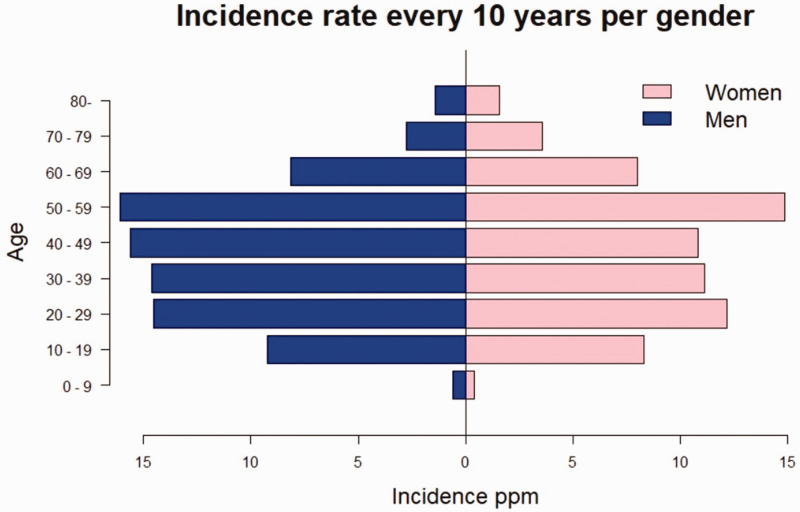
Mean annual crude incidence rate of Kienböck’s disease in Finland in 10 year age groups by sex; ppm = patients per one million person-years.

There were 759 (54%) men and 635 (46%) women in our study population. Men had the higher incidence in all age groups up to 60–69 years. In women, as in the total population, the incidence peak was bimodal, at 20–29 and 50–59 years. For men, the highest incidence rates were in middle age, between 40 and 59. The incidence rates by sex and age group are shown in Figure 1. Based on the incidence rate, the men to women ratio in our data was 1.24:1, and the median age at diagnosis was 42 years for women and 41 years for men. The age range for women was 6–87 and for men it was 6–81 years.

**Figure 2.**
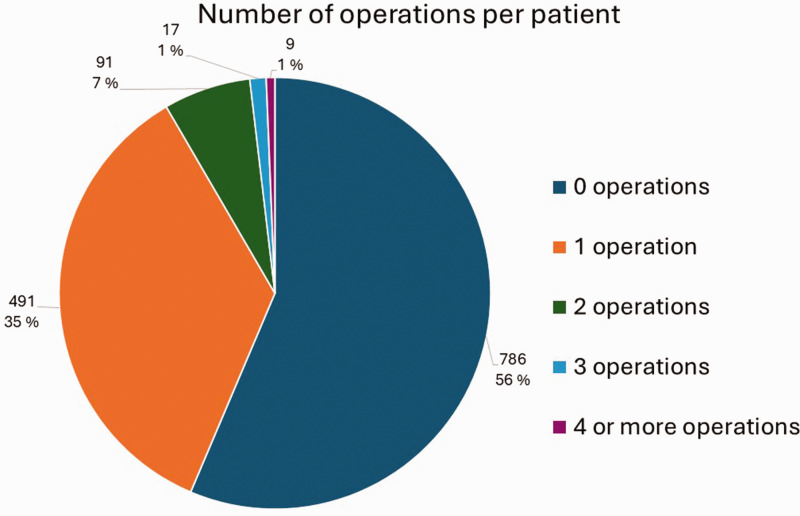
The number of times patients were operated on for Kienböck’s Disease. The percentages shown are the proportions of the total amount of patients.

**Figure 3.**
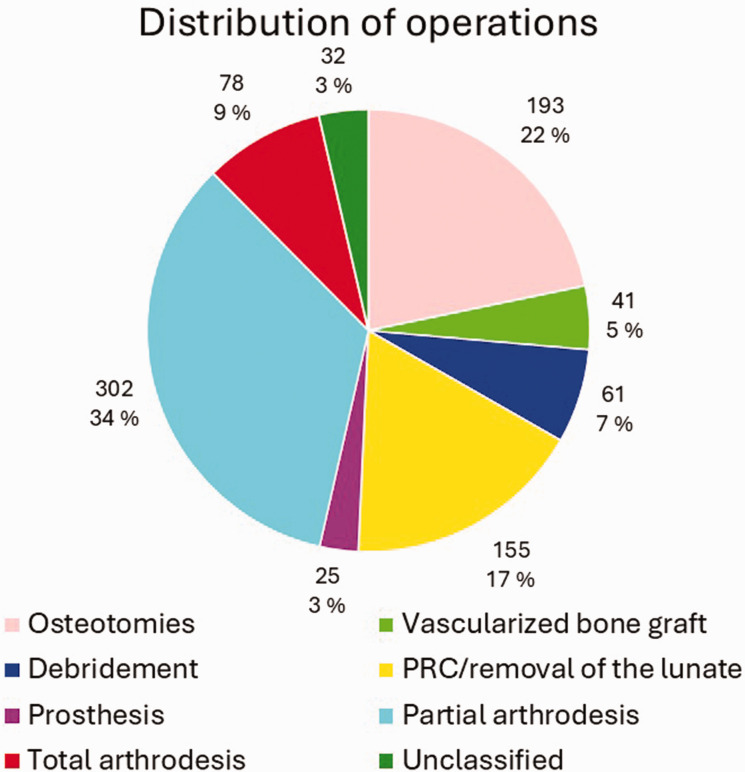
Distribution of surgical procedures according to previously decided classes (Table 1) in our data.

**Figure 4.**
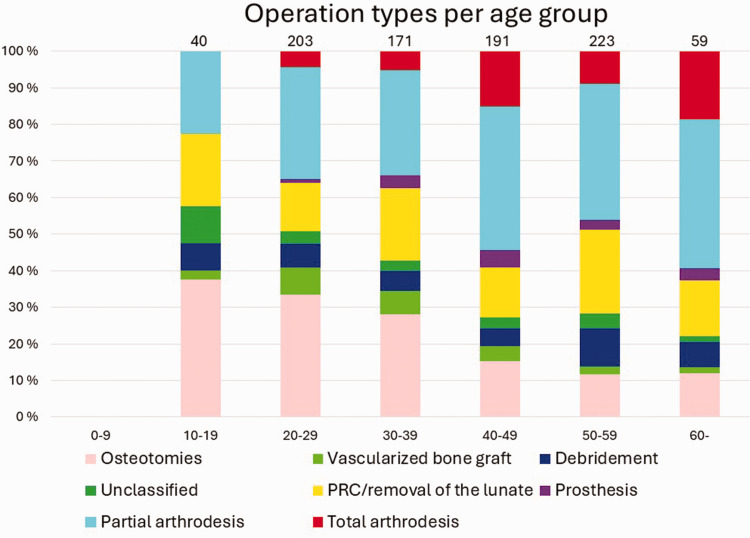
Distribution of different surgical procedures performed per age group every 10 years. Numbers on top of the bars are total numbers of operations per age group.

Altogether 608 patients (44%) had undergone at least one operation for KD, and 117 of them (19%) had been operated on more than once. Figure 2 presents the number of operations per patient. The highest number of operations per patient was six. Of all the operated patients, 353 (58%) were men and 255 (42%) were women. In our study, six of the 92 (6.5%) 0–15-year-old, 48% of the 16–69-year-old and six of the 48 (13%) over 70-year-old KD patients had been operated on. The bimodal peak of incidence in the total population most likely accounts for the highest number of operations in 20–29-year-olds and 50–59-year-olds. Compared with other procedure types, osteotomies and vascularized bone grafts were more common among the 10–39-year-old patients, whereas PWA and TWA seemed to be preferred for the middle-aged and older patients. Proximal row carpectomy/removal of the lunate was done quite evenly across all the age groups. Prostheses were mainly performed in the 30–59-year-olds. Salvage procedures accounted for half of the procedures in 20–29-year-olds, and for the older patients most were salvage procedures. Figure 3 shows the distribution of the different surgical procedure types across the patients. Figure 4 shows this in the age groups. Altogether 62% of all the procedures were classified as salvage procedures and 54% of these were done in men. The male proportion of non-salvage procedures was 59%. The distribution to salvage and non-salvage procedures was not statistically significantly different between men and women (*p* = 0.20).

Lastly, we observed some combinations of different operations in our data. In total, 15 of the 243 (6.2%) patients who underwent PWA later ended up having TWA. However, 45 of the 243 (19%) PWA patients had some salvage procedure afterwards, either revision or some other salvage procedure. Out of the 41 patients who underwent debridement, 17 (41%) also underwent a further operation. Of the patients who received vascularized bone grafts, seven out of 40 (18%) later had some salvage procedure. Among the patients with osteotomies, the corresponding number was 16 out of 171 (9.3%). Table 2 shows the number and percentage of patients who ended up having TWA after different surgical procedures.

**Table 2.** Number and percentage of patients who ended up having total wrist arthrodesis after different surgical procedures during the study period, 1996–2022.

Operation	Number of total wrist arthrodeses	Total operated patients	Percentage (%)
Osteotomies	5	171	2.9
Debridement	8	41	20
PRC	11	96	11
Partial wrist arthrodesis	15	243	6.2

PRC, Proximal row carpectomy.

## Discussion

Our study found that the total mean annual standardized incidence of KD was 9.60 ppm in Finland in 1996–2022. The incidence rate was higher among men than women, with the 50–59-year-old group being the most affected by KD.

Previous studies have primarily focused on reporting the prevalence of incidental findings in cross-sectional settings. Van Leeuwen and colleagues studied a large cohort of 51,071 patients over 11 years and found the total prevalence to be 0.27%, of which the prevalence of asymptomatic cases was 0.10% (van Leeuwen et al., 2016). The equivalent prevalences of symptomless KD were reported as 0.0066% in a UK population of 76,174 patients (Golay et al., 2016) and 1.9% in an African population of 1287 patients (Mennen and Sithebe, 2009). The reported prevalences vary notably, probably caused by selection bias in these studies. These rates are not entirely comparable with our own results because we studied the incidence of the entire population and not the period prevalence of asymptomatic cases. However, our nationwide incidence rates concur with previous claims and reports that KD is rare.

Previous studies have described higher female predominance in the older population (Taniguchi et al., 2003). This aligns with our finding that the incidence rate for men was higher in all age groups except the over 70-year-olds. However, the male predominance in our study, based on the incidence rate of 1.24:1 (54% of all cases), is not as high as previous research has suggested (White et al., 2016). Our study results also seem to challenge previous dogma that KD mostly affects patients aged between 20 and 40 (Rioux–Forker and Shin, 2020; Schuind et al., 2008), as the total and sex-based incidence rates were highest at the age of 50–59 in our study.

A total of 44% of the patients were operated on for KD at least once, and 19% of them were operated on twice or more. Reconstructive procedures seemed to be favoured for younger patients, while the proportion of salvage procedures increased with age. We did not have access to the stages of KD, so we can only speculate whether this trend was due to the presence of earlier stages in younger patients or because surgeons wanted to avoid salvage procedures for them. In our study, 62% of all the operations were salvage procedures. The large proportion of salvage procedures may be caused by delayed diagnosis with more advanced disease, limiting the available surgical procedures. The incidence peak at an older age (50–59 years) in our study may also be partially because of delayed diagnosis.

Usually, scaphotrapeziotrapezoid or scaphocapitate arthrodesis are the PWAs opted for KD when the lunate is no longer salvageable. In their systematic review, Stephens and colleagues (2022) found arthrodesis fairly good results for scaphotrapeziotrapezoid, but a relatively high non-union rate of 14.1%. A recent systematic review and meta-analysis reported that pain diminished after scaphocapitate arthrodesis, but the overall complication and reoperation rates were high, 24 and 12%, respectively (Bouri et al., 2023). Reports on the results of PRC in the treatment of KD are solely from case reports with variable complication rates, and the risk of total arthrodesis varies from 0 to 33% (Bijon et al., 2020; Buluç et al., 2015; Lumsden et al., 2008). In our data, a higher proportion of patients ended up with TWA after PRC than after PWA, 11 out of 96 (11%) and 15 out of 243 (6.2%), respectively. However, the fairly high revision/salvage rate of 45 out of 243 (19%) for PWAs in our data suggests that different alternatives could be considered. For example, Yamamoto et al. (2021) recently showed that osteotomies resulted in significant clinical improvement, even for the older group of patients over 40 years. Previous research has also suggested that the preoperative Lichtman classification is not a significant factor in clinical outcome after radial osteotomies (Iwasaki et al., 2003; Nakamura et al., 1990). The conversion to TWA was also the lowest for osteotomies in our data: five out of 171 (2.9%).

The strength of this study is that we used a reliable, nationwide register that covers the whole population of Finland (Sund, 2012). A limitation of register studies is that diagnoses cannot be confirmed from patient charts, and there is always the risk of inaccurate recordings. In particular, suspicions of KD could be documented as being true KD (Sund, 2012). Surgical procedures were coded according to the Finnish version of the Nordic Classification of Surgical Procedures. Our study is thus limited by the classification and some more generic procedure codes may include many different types of procedures. Accordingly, our distribution of different procedures into classes may be somewhat arbitrary. The laterality of the disease or operation is not available in the register. Hence, it is impossible to know whether some patients have a bilateral disease or revision surgery on the same side. The operated patients’ KD stage is unknown, and the discussion on this is very hypothetical.

There were also some obscurities regarding the recordings of some of the procedure codes under the same operation. We decided to report the number of all the procedures presented in the data we received, as we wanted to avoid creating additional bias. They may present as a biased high number in certain operation classes, but these were excluded from the operation combination calculations. For these numbers, we assumed the main procedure in each operation according to the prioritizing command explained in our methods. A considerable number of patients in our study did not undergo any operation. We did not analyse these in more detail because the register data possibly had false positive diagnoses and thus could lead to false conclusions.

Based on our research, KD is a rare disease that mostly affects working-age patients. In contrast to the findings of previous studies, 50–59-year-olds were the most affected and male predominance was not as high as previously reported. Salvage procedures were performed unexpectedly often, and reoperations were common, which underlines the fact that this disease may have significant consequences for patients and society. More research is needed to determine which patients are at the most risk of developing KD with a poorer prognosis, to target their treatment appropriately and in time.

## References

[bibr1-17531934241312894] Bijon C Saab M Amouyel T Sturbois-Nachef N Guerre E Chantelot C. Long-term radiological changes and functional outcomes after proximal row carpectomy: Retrospective study with 3 years’ minimum follow-up. Orthop Traumatol Surg Res. 2020, 106: 1589–95. 33289656 10.1016/j.otsr.2020.03.038

[bibr2-17531934241312894] Bouri F Hantouly AT Alzobi O , et al. Clinical and radiological outcomes of scaphocapitate fusion in Kienböck disease: A systematic review and meta-analysis. J Hand Surg Glob Online. 2023, 5: 435–44. 37521555 10.1016/j.jhsg.2023.03.014 PMC10382890

[bibr3-17531934241312894] Buluç L Gündes H Baran T Selek Ö. Proximal row carpectomy for Lichtman stage III Kienböck’s disease. Acta Orthop Traumatol Turc. 2015, 49: 641–7. 26511691 10.3944/AOTT.2015.14.0346

[bibr4-17531934241312894] Daly CA Graf AR. Kienböck disease: Clinical presentation, epidemiology, and historical perspective. Hand Clin. 2022, 38: 385–92. 36244706 10.1016/j.hcl.2022.03.002

[bibr5-17531934241312894] Degeorge BR Chawla SS Lewallen L Kakar S. Functional and radiographic disease progression in nonoperatively managed Kienböck disease. Plast Reconstr Surg. 2021, 147: 1117–23. 33890893 10.1097/PRS.0000000000007838

[bibr6-17531934241312894] Golay SK Rust P Ring D. The radiological prevalence of incidental Kienböck disease. Arch Bone Jt Surg. 2016, 4: 220–3. 27517065 PMC4969366

[bibr7-17531934241312894] Ho Shin Y Kwang Kim J Han M Kyoon Lee T Yoon JO. Comparison of long-term outcomes of radial osteotomy and nonoperative treatment for Kienböck disease: A systematic review. J Bone Joint Surg Am. 2018, 100: 1231–40. 30020130 10.2106/JBJS.17.00764

[bibr8-17531934241312894] Hwang JS Shim BJ Li Q Kim J Baek GH. The natural history of Kienböck’s disease diagnosed at more than 50 years of age. Clin Orthop Surg. 2022, 14: 450–7. 36061838 10.4055/cios22022 PMC9393282

[bibr9-17531934241312894] Innes L Strauch RJ. Systematic review of the treatment of Kienböck’s disease in its early and late stages. J Hand Surg Am. 2010, 35: 713–7. 20438990 10.1016/j.jhsa.2010.02.002

[bibr10-17531934241312894] Irisarri C Kalb K Ribak S. Infantile and juvenile lunatomalacia. J Hand Surg Eur. 2010, 35: 544–8. 10.1177/1753193410364913 20237187

[bibr11-17531934241312894] Iwasaki N Minami A Oizumi N Yamane S Suenaga N Kato H. Predictors of clinical results of radial osteotomies for Kienböck’s disease. Clin Orthop Relat Res. 2003, 415: 157–62. 10.1097/01.blo.0000093907.26658.3b 14612642

[bibr12-17531934241312894] Kristensen SS Thomassen E Christensen F. Kienböck’s disease—Late results by non-surgical treatment: A follow-up study. J Hand Surg Br. 1986, 11: 422–5. 3794489 10.1016/0266-7681(86)90171-3

[bibr13-17531934241312894] van Leeuwen WF Janssen SJ ter Meulen DP Ring D. What is the radiographic prevalence of incidental Kienböck disease? Clin Orthop Relat Res. 2016, 474: 808–13. 26324836 10.1007/s11999-015-4541-1 PMC4746165

[bibr14-17531934241312894] Lichtman DM Pientka WF II Bain GI. Kienböck disease: A new algorithm for the 21st century. J Wrist Surg. 2017, 6: 2–10. 28119790 10.1055/s-0036-1593734 PMC5258126

[bibr15-17531934241312894] Lumsden BC Stone A Engber WD. Treatment of advanced-stage Kienböck’s disease with proximal row carpectomy: An average 15-year follow-up. J Hand Surg Am. 2008, 33: 493–502. 18406952 10.1016/j.jhsa.2007.12.010

[bibr16-17531934241312894] Martin GR Squire D. Long-term outcomes for Kienböck’s disease. Hand (NY). 2013, 8: 23–6. 10.1007/s11552-012-9470-9 PMC3574490 24426889

[bibr17-17531934241312894] Martini AK. Der spontane verlauf der Lunatummalazie [The spontaneous course of lunate malacia]. Handchir Mikrochir Plast Chir. 1990, 22: 14–9. 2311992

[bibr18-17531934241312894] Mennen U Sithebe H. The incidence of asymptomatic Kienböck’s disease. J Hand Surg Eur. 2009, 34: 348–50. 10.1177/1753193408098481 19457902

[bibr19-17531934241312894] Nakamura R Imaeda T Miura T. Radial shortening for Kienböck’s disease: Factors affecting the operative result. J Hand Surg Br. 1990, 15: 40–5. 2307879 10.1016/0266-7681_90_90045-6

[bibr20-17531934241312894] Park JY Kim JK Shin YH. Comparison of long-term outcomes between nonoperative treatment and vascularized bone graft for Kienböck disease: A systematic review and single-arm meta-analysis. Clin Orthop Surg. 2023, 15: 643–52. 37529196 10.4055/cios22307 PMC10375810

[bibr21-17531934241312894] Rioux-Forker D Shin AY. Osteonecrosis of the lunate: Kienböck disease. J Am Acad Orthop Surg. 2020, 28: 570–84. 32692092 10.5435/JAAOS-D-20-00020

[bibr22-17531934241312894] Schuind F Eslami S Ledoux P. Kienböck’s disease. Bone Joint Surg Br. 2008, 90: 133–139. 10.1302/0301-620X.90B2.20112 18256076

[bibr23-17531934241312894] Stahl S Hentschel PJH Held M , et al. Characteristic features and natural evolution of Kienböck’s disease: Five years’ results of a prospective case series and retrospective case series of 106 patients. J Plast Reconstr Aesthet Surg. 2014, 67: 1415–26. 24947083 10.1016/j.bjps.2014.05.037

[bibr24-17531934241312894] Stephens AR Garcia BN Rogers MJ , et al. Scaphotrapeziotrapezoid arthrodesis: Systematic review. J Hand Surg Am. 2022, 47: 218–27. 35033404 10.1016/j.jhsa.2021.09.029

[bibr25-17531934241312894] Sund R. Quality of the Finnish hospital discharge register: A systematic review. Scand J Public Health. 2012, 40: 505–15. 22899561 10.1177/1403494812456637

[bibr26-17531934241312894] Taniguchi Y Yoshida M Iwasaki H Otakara H Iwata S. Kienböck’s disease in elderly patients. J Hand Surg Am. 2003, 28: 779–83. 14507507 10.1016/s0363-5023(03)00299-5

[bibr27-17531934241312894] Tsujimoto R Maeda J Abe Y , et al. Epidemiology of Kienböck’s disease in middle-aged and elderly Japanese women. Orthopedics. 2015, 38: e14–e18. 25611414 10.3928/01477447-20150105-54

[bibr28-17531934241312894] Van den Dungen S Dury M Foucher G Marin Braun F Loréa P. Conservative treatment versus scaphotrapeziotrapezoid arthrodesis for Kienbock’s disease. A retrospective study. Chir Main. 2006, 25: 141–5. 17175800 10.1016/j.main.2006.07.030

[bibr29-17531934241312894] Viljakka T Tallroth K Vastamäki M. Long-term natural outcome (7–26 years) of Lichtman stage III Kienböck’s lunatomalacia. Scand J Surg. 2016, 105: 125–32. 25862717 10.1177/1457496915577023

[bibr30-17531934241312894] Wang PQ Matache BA Grewal R Suh N. Treatment of stages IIIA and IIIB in Kienbock’s disease: A systematic review. J Wrist Surg. 2020, 9: 535–48. 33282541 10.1055/s-0040-1716353 PMC7708034

[bibr31-17531934241312894] White C Benhaim P Plotkin B. Treatments for Kienböck disease: what the radiologist needs to know. Skeletal Radiol. 2016, 45: 531–40. 26802001 10.1007/s00256-016-2332-8

[bibr32-17531934241312894] Yamamoto M Tatebe M Nakagawa Y Kurimoto S Iwatsuki K Hirata H. Radial osteotomy for Kienböck disease: Clinical and radiological comparison between younger and older patients. J Hand Surg Asian Pac Vol. 2021, 26: 410–16. 34380386 10.1142/S2424835521500405

